# Magneto-optical design of anomalous Nernst thermopile

**DOI:** 10.1038/s41598-021-90865-5

**Published:** 2021-05-27

**Authors:** Jian Wang, Asuka Miura, Rajkumar Modak, Yukiko K. Takahashi, Ken-ichi Uchida

**Affiliations:** 1 National Institute for Materials Science, Tsukuba, 305-0047 Japan; 2 Institute for Materials Research, Tohoku University, Sendai, 980-8577 Japan; 3 Center for Spintronics Research Network, Tohoku University, Sendai, 980-8577 Japan; 4 Present Address: Magnetic Powder Metallurgy Research Center, National Institute of Advanced Industrial Science and Technology, Nagoya, 463-8560 Japan; 5 Present Address: Integrated Research for Energy and Environment Advanced Technology, Kyushu Institute of Technology, Kitakyushu, Fukuoka 804-8550 Japan

**Keywords:** Energy harvesting, Magnetic properties and materials, Spintronics

## Abstract

The introduction of spin caloritronics into thermoelectric conversion has paved a new path for versatile energy harvesting and heat sensing technologies. In particular, thermoelectric generation based on the anomalous Nernst effect (ANE) is an appealing approach as it shows considerable potential to realize efficient, large-area, and flexible use of heat energy. To make ANE applications viable, not only the improvement of thermoelectric performance but also the simplification of device structures is essential. Here, we demonstrate the construction of an anomalous Nernst thermopile with a substantially enhanced thermoelectric output and simple structure comprising a single ferromagnetic material. These improvements are achieved by combining the ANE with the magneto-optical recording technique called all-optical helicity-dependent switching of magnetization. Our thermopile consists only of Co/Pt multilayer wires arranged in a zigzag configuration, which simplifies microfabrication processes. When the out-of-plane magnetization of the neighboring wires is reversed alternately by local illumination with circularly polarized light, the ANE-induced voltage in the thermopile shows an order of magnitude enhancement, confirming the concept of a magneto-optically designed anomalous Nernst thermopile. The sign of the enhanced ANE-induced voltage can be controlled reversibly by changing the light polarization. The engineering concept demonstrated here promotes effective utilization of the characteristics of the ANE and will contribute to realizing its thermoelectric applications.

## Introduction

Thermoelectric conversion can be a key technology for versatile energy harvesting and thermal management applications^1,2^. Thermoelectric generators allow the generation of electrical voltage or power from thermal energy, offering a unique solution to sustainable power generation from waste heat^3,4^. Currently, there is an increasing demand for novel energy harvesting technologies that can efficiently generate electricity to power Internet-of-Things sensors and increasingly complex electronic devices^5,6^. The existing thermoelectric generators are based on the longitudinal thermoelectric effect, i.e., the Seebeck effect (SE), which converts a temperature gradient ∇*T* to an electric field in a conductor in the direction parallel to ∇*T*.

As a principle for developing next-generation thermoelectric generators, the transverse thermoelectric effect called the anomalous Nernst effect (ANE) has attracted increasing interest owing to its unique symmetry and convenient scaling behaviors^7–22^. Here, the ANE refers to the generation of an electric field in a magnetic material along the outer product of ∇*T* and magnetization **M**. The electric field driven by the ANE (**E**_ANE_) is described as

1 $${\mathbf{E}}_{{{\text{ANE}}}} = S_{{{\text{ANE}}}} \left( {\frac{{\mathbf{M}}}{{\left| {\mathbf{M}} \right|}}} \right) \times \nabla T$$

where *S*_ANE_ is the anomalous Nernst coefficient. With such a unique symmetry, the ANE enables efficient and flexible use of various heat sources over a large non-flat surface. However, the reported thermoelectric output of the ANE is much lower than that of the SE. Thus, to utilize the unique advantages of the ANE for thermoelectric applications, it is necessary to develop a novel approach to achieve a high thermoelectric output.

**Figure 1:**
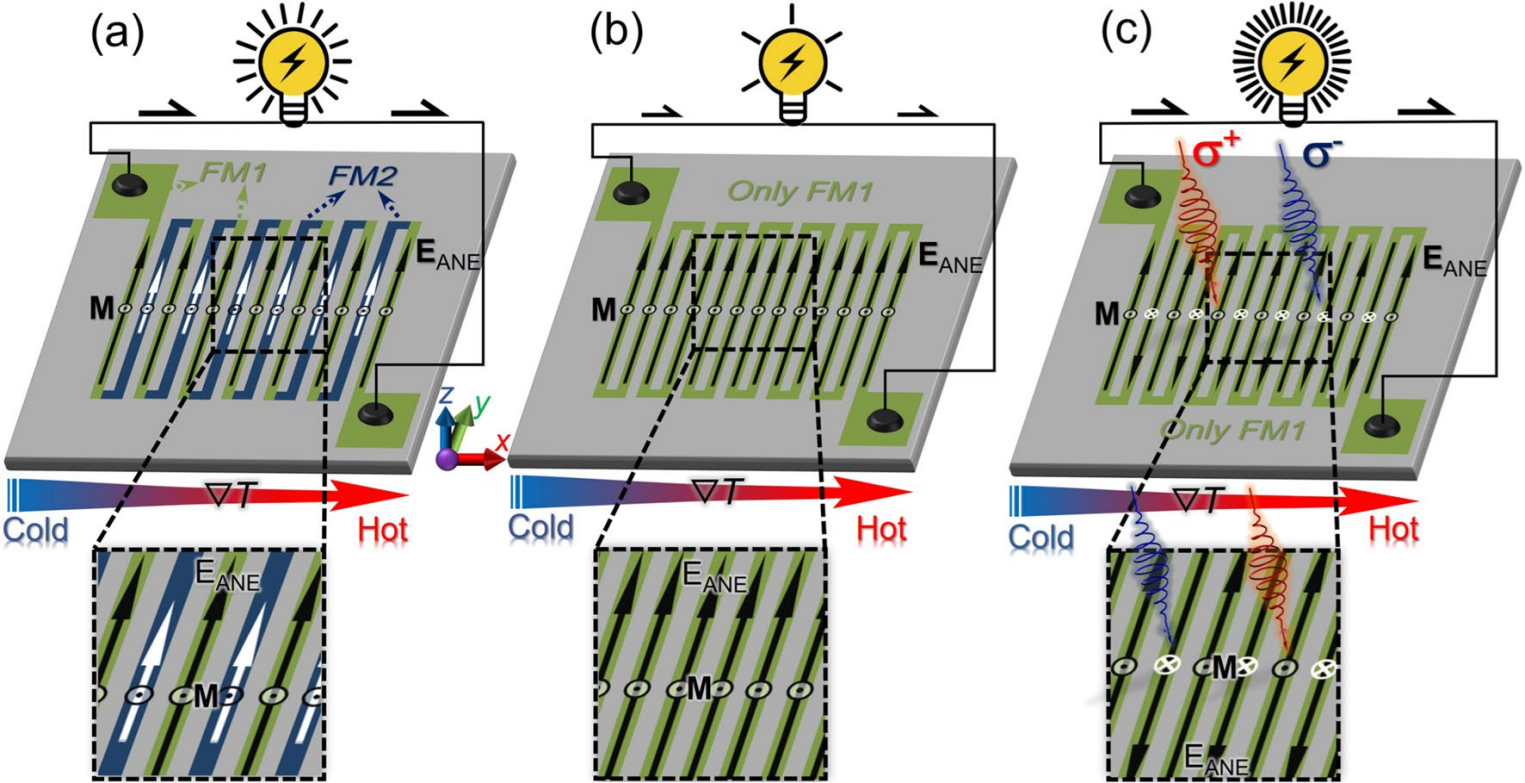
**(a)** Schematic of the anomalous Nernst thermopile consisting of two ferromagnetic materials (FM1 and FM2) with different anomalous Nernst coefficients. Here, **E**_ANE_ stands for the electric field driven by the ANE, **M** the magnetization, and ∇*T* the temperature gradient. **(b)** Schematic of the zigzag structure consisting of a single ferromagnetic material with uniform out-of-plane magnetization. **(c)** Schematic of the anomalous Nernst thermopile consisting of a single ferromagnetic material with alternately reversed out-of-plane magnetization realized by all-optical helicity-dependent switching of magnetization. **σ**^+^ (**σ**^**−**^) indicates right (left) circularly polarized light.

Recent works have demonstrated that the thermoelectric voltage induced by the ANE can be dramatically enhanced by constructing a simple lateral thermopile structure, whereas the thermopile for the SE has a complex three-dimensional structure^18,22^. A conventional anomalous Nernst thermopile consists of laterally connected thermocouples comprising two different ferromagnetic metal wires in a zigzag configuration (Fig. 1a). If the two connected ferromagnetic wires have different sign or magnitude of *S*_ANE_ and/or different coercive forces, the thermoelectric voltage generated from each wire can be added to the total output voltage in series (Fig. 1a). However, the complicated microfabrication processes and contact resistances of thermopile structures involving two different materials hinder the merits of the ANE. In contrast, if a zigzag structure is developed using only a single material and each wire is magnetized uniformly, most of the thermoelectric voltage is cancelled out, which does not work as a thermopile (Fig. 1b).

In this study, we have demonstrated the construction of an anomalous Nernst thermopile consisting only of a single perpendicularly magnetized material with the aid of magneto-optical effects. To avoid the aforementioned cancellation of the thermoelectric voltage between the neighboring wires, their **M** direction should be reversed alternately, allowing us to boost the voltage in the simple zigzag structure (Eq. (1) and Fig. 1c). Although such a magnetization configuration cannot be achieved in a single material by applying an external magnetic field, we overcome this difficulty using all-optical helicity-dependent switching (AO-HDS) of magnetization^23–28^. The AO-HDS refers to deterministic magnetization switching in magnetic materials as a result of circularly polarized light illumination, where the **M** orientation is determined by the light helicity. As shown in Fig. 1c, with a perpendicularly magnetized zigzag-shaped ferromagnetic wire series, one can reverse the **M** direction of the selected wires via the AO-HDS by locally illuminating the wires with circularly polarized light without applying external magnetic fields. By repeating this process, a configuration with alternately reversed magnetization can be established, thereby adding the ANE voltages in all the wires to the total output voltage in series. The total thermoelectric voltage can be proportionally enhanced by elongating the total wire length, i.e., increasing the number of the wires and/or the length of each wire. Compared to conventional thermoelectric devices based on the SE, the proposed anomalous Nernst thermopile can substantially reduce the number of fabrication processes and the contact resistance in the circuit. This can pave the way for developing thermoelectric devices with high flexibility, high mechanical endurance, low thermal resistance, and low production costs. It is also worth noting that the output ANE voltage achieved using the proposed approach is programable or tunable in sign and magnitude through the appropriate design of the magnetization configuration via the AO-HDS. For example, the sign of the total ANE voltage in the thermopile can be reversibly changed by switching the **M** direction of each wire using circularly polarized light having the opposite helicity. Additionally, the magnitude of the output ANE voltage can be varied by programing the number of wires magnetically switched via the AO-HDS.

## Results and discussion

### Magnetic and magneto-optical properties

**Figure 2:**
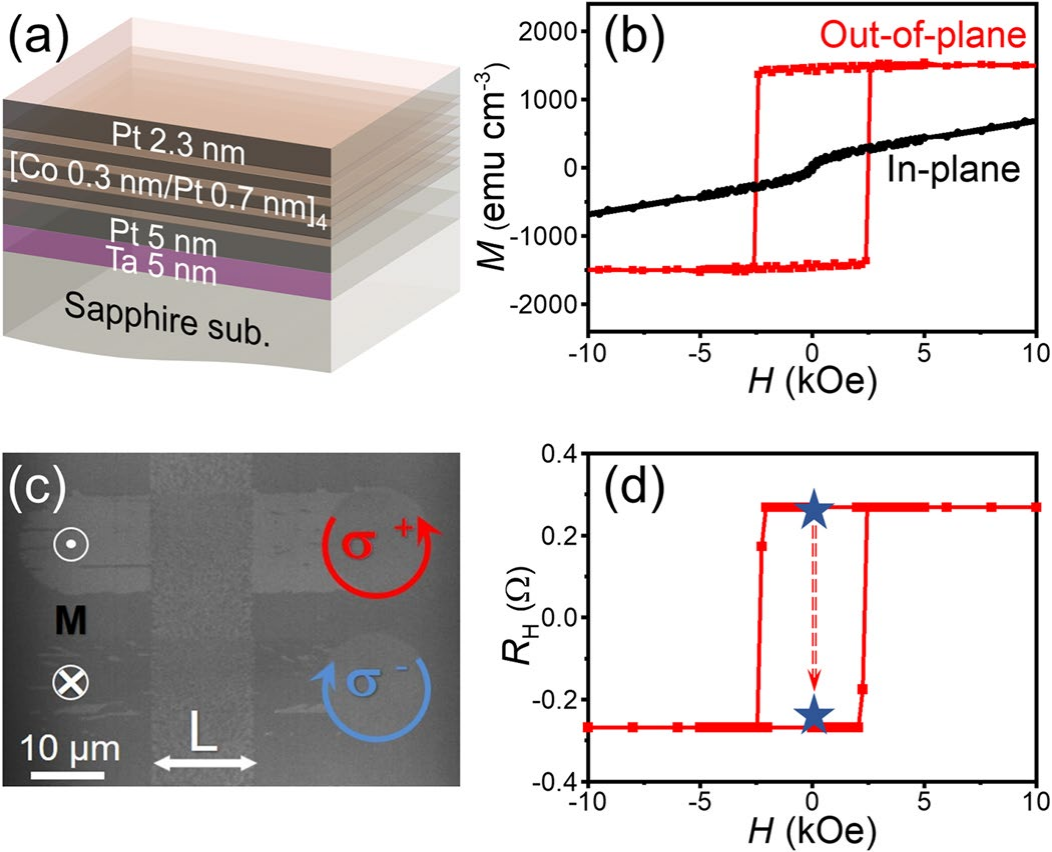
**(a)** Layer configuration of the [Co/Pt]_4_ film. **(b)** Magnetization *M* curves of the [Co/Pt]_4_ film, measured when a magnetic field *H* was applied along the easy axis (out-of-plane direction of the film) and the hard axis (in-plane direction of the film) at room temperature. The [Co/Pt]_4_ film exhibits strong perpendicular magnetic anisotropy with an out-of-plane coercive field of 2.5 kOe and an anisotropy field of 25.1 kOe. **(c)** MOKE image of the [Co/Pt]_4_ film illuminated with right (**σ**^+^) and left (**σ**^**−**^) circularly polarized laser light as well as linearly (**L**) polarized laser light at room temperature. The bright (dark) contrast represents the area with **M** along the + *z* (− *z*) direction perpendicular to the film plane. **(d)** Hall resistance *R*_H_ of the [Co/Pt]_4_ film with a Hall bar shape at room temperature. The red line shows the out-of-plane *H* dependence of *R*_H_. The blue stars indicate the *R*_H_ values before and after illuminating the entire Hall bar with **σ**^**−**^ light at zero field, where the Hall bar was uniformly magnetized by applying an external field *H* =  + 20 kOe before light illumination.

To demonstrate the magneto-optical design of the anomalous Nernst thermopile, we employed a Ta (5)/Pt (5)/[Co (0.3)/Pt (0.7)]_4_/Pt (2.3) multilayer film, where the numbers in parentheses indicate the layer thicknesses in nanometers and the subscript indicates the stacking number (see Fig. 2a and “Methods” for details). The Co/Pt multilayer film is known to exhibit the AO-HDS^25–28^. Hereafter, the film is referred to as the [Co/Pt]_4_ film for simplicity. The squared out-of-plane magnetization curve in Fig. 2b indicates a strong perpendicular magnetic anisotropy with an out-of-plane coercivity of approximately 2.5 kOe for the [Co/Pt]_4_ film. Figure 2c shows a magneto-optic Kerr effect (MOKE) microscope image of the [Co/Pt]_4_ film obtained after sweeping with right (**σ**^+^) and left (**σ**^**−**^) circularly polarized laser light as well as linearly (**L**) polarized light. The dark contrast in the MOKE image represents the magnetic domain with the magnetic moments pointing into the film plane, whereas the bright contrast represents the magnetic moments pointing out of the film plane. Since the linearly polarized light contains of equal summation of right and left circularly polarized light, it leaves a multi-domain trace with average domain size ~ 500 nm. The distinct magneto-optical responses depending on the light polarization clearly indicates the AO-HDS in our [Co/Pt]_4_ film. The light-induced magnetization reversal was indirectly quantified via the anomalous Hall effect with a microfabricated Hall-cross device (see Fig. 2d and Methods for details). The estimated AO-HDS efficiency was approximately 91%, which is sufficiently high to ensure a suitable platform for demonstrating the magneto-optical control of the ANE-induced thermoelectric voltage.

**Figure 3:**
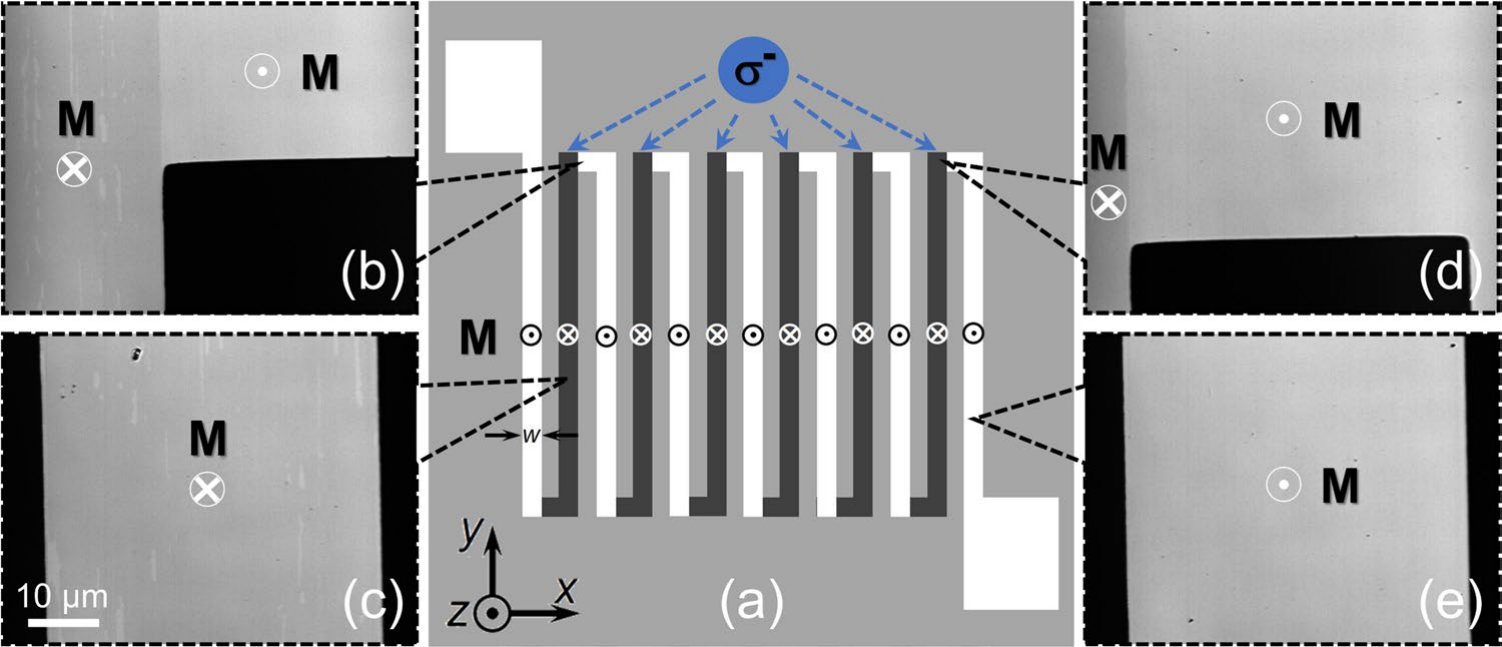
**(a)** Schematic of the zigzag-shaped [Co/Pt]_4_ film with the alternately reversed magnetization configuration, where the **M** direction is reversed between the neighboring wires. **(b–e)** Representative local MOKE images of the zigzag-shaped [Co/Pt]_4_ film. The magnetization configuration was designed by alternately irradiating the wires with left (**σ**^**−**^) circularly polarized laser light, where **M** was along the + *z* direction before light illumination. The bright (dark) contrast in the MOKE images represents **M** along to the + *z* (− *z*) direction.

For the thermoelectric voltage measurements, the [Co/Pt]_4_ film was microfabricated into a zigzag shape with 13 individual wires (Fig. 3a). Then, as illustrated in Fig. 3a, the alternately reversed magnetization configuration between the adjacent wires was realized via the AO-HDS by sweeping the perpendicular magnetized wires with circularly polarized light. Figure 3b–e show the representative MOKE images obtained from specific regions of the zigzag structure, which prove the effectiveness of the magnetization configuration designed using the AO-HDS. Furthermore, owing to pinning of the magnetic domain walls to the defect sites of the [Co/Pt]_4_ film, there are fractional unreversed regions after laser sweeping, which can be visualized as tiny traces (areas with brighter contrast) in Fig. 3b,c. This is responsible for the observed AO-HDS efficiency of approximately 91% (not 100%).

### Separation of ANE from Seebeck effect

**Figure 4:**
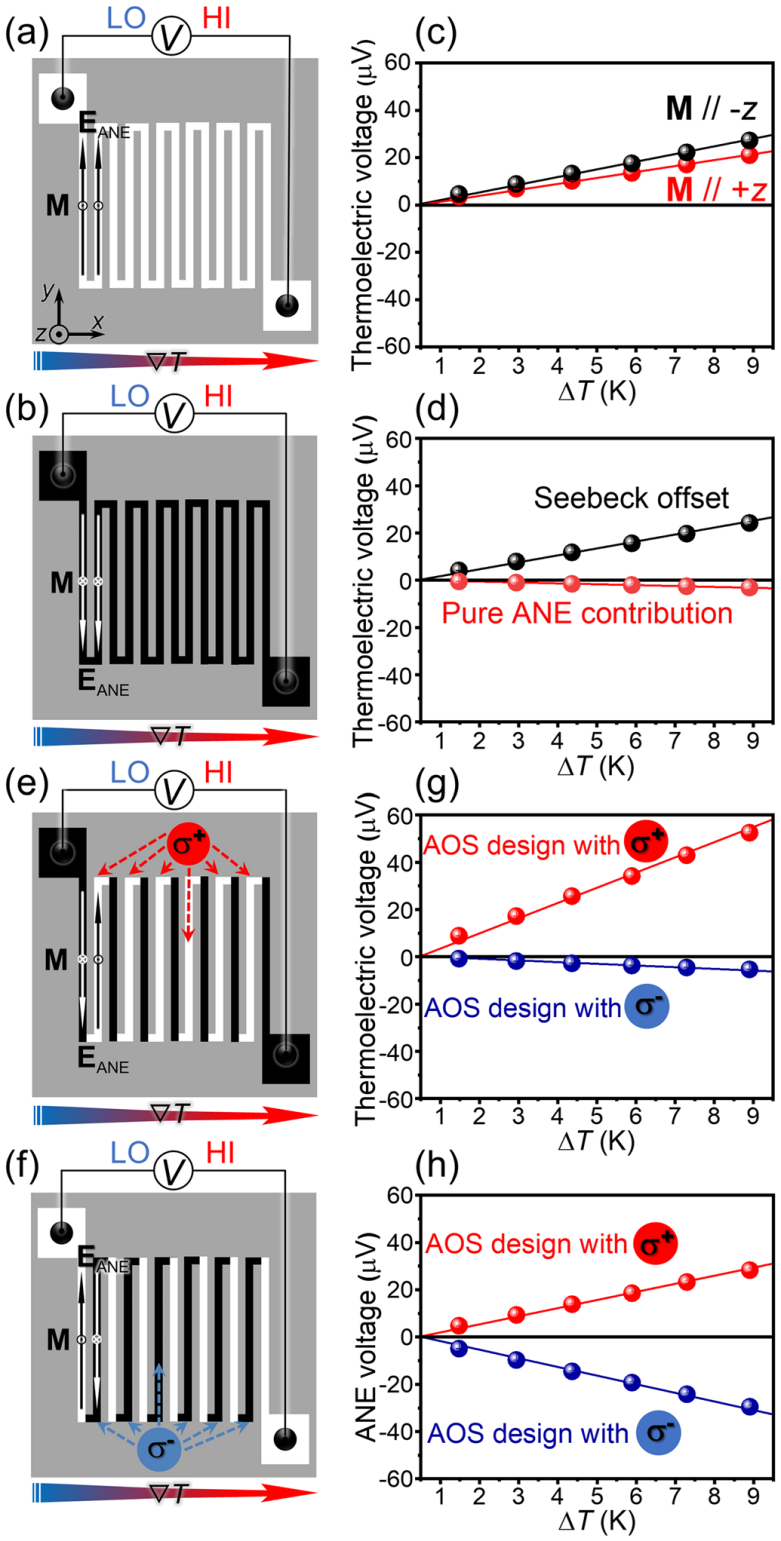
**(a,b)** Schematics of the zigzag-shaped [Co/Pt]_4_ film uniformly magnetized along the + *z* **(a)** and − *z* **(b)** directions. **(c)** Temperature difference Δ*T* dependence of thermoelectric voltage in the zigzag-shaped [Co/Pt]_4_ film uniformly magnetized along the − *z* or + *z* direction. **(d)** Δ*T* dependence of *V*_ANE_ (= (*V*_+_ – *V*_–_)/2) and *V*_SE_ (= (*V*_+_  + *V*_–_)/2), calculated from the data in **(c)**. *V*_+_ (*V*_–_) denotes the thermoelectric voltage measured when **M** is along the + *z* (− *z*) direction. **(e,f)** Schematics of the zigzag-shaped [Co/Pt]_4_ film in the alternately reversed magnetization configurations designed via the AO-HDS. The configuration in **(e)** (**(f)**) was designed by irradiating the wires uniformly magnetized along the − *z* (+ *z*) direction with right (**σ**^**+**^) (left (**σ**^**−**^)) circularly polarized light. **(g)** Δ*T* dependence of the thermoelectric voltage in the zigzag-shaped [Co/Pt]_4_ film in the alternately reversed magnetization configurations. **(h)** Δ*T* dependence of *V*_ANE_ in the zigzag-shaped [Co/Pt]_4_ film in the alternately reversed magnetization configurations, calculated by subtracting the SE background in **(d)** from the raw data in **(g)**.

We then investigated the thermoelectric properties of the zigzag [Co/Pt]_4_ film with the designed magnetization pattern. Figure 4a,b,e,f illustrates the geometrical relationships among the designed magnetization configuration, applied temperature gradient (∇*T*), and **E**_ANE_ in the zigzag structure. To extract the pure ANE contribution in the [Co/Pt]_4_ film, it is necessary to estimate the inevitable background voltage resulting from the SE. This background voltage can be estimated by comparing the thermoelectric voltage, *V*, in the [Co/Pt]_4_ film uniformly magnetized along the − *z* direction with that along the + *z* direction as the SE (ANE) voltage exhibits the no or even (odd) dependence on the **M** reversal. To do this, the film was uniformly magnetized by applying an external magnetic field of ~ 4 kOe before the *V* measurements (note that *V* was measured in the absence of the magnetic field). The ANE and SE contributions were thus obtained from *V*_ANE_ = (*V*_+_ – *V*_–_)/2 and *V*_SE_ = (*V*_+_  + *V*_–_)/2, respectively, where *V*_+_ (*V*_–_) denotes *V* measured when **M** is uniformly aligned in the + *z* (− *z*) direction. We carefully performed the *V* measurements using the identical [Co/Pt]_4_ film with various magnetization configurations so that the magnitude of *V*_SE_ remained the same in all the measurements. Figure 4c shows the measured *V*_–_ and *V*_+_ values for the uniformly magnetized [Co/Pt]_4_ film as a function of the temperature difference, Δ*T*, between the voltage terminals. We found that *V*_+_ and *V*_–_ were proportional to Δ*T* and the magnitude of *V*_–_ was higher than that of *V*_+_ at each Δ*T* value, suggesting the presence of *V*_ANE_. Using the above definition, we obtained the Δ*T* dependence of *V*_ANE_ and *V*_SE_ for the uniformly magnetized [Co/Pt]_4_ film; *V*_SE_ was one order of magnitude higher than *V*_ANE_ in this configuration (Fig. 4d). By linearly fitting the experimental data, the ANE (SE) thermopower was estimated as *V*_ANE_/Δ*T* = –0.35 µV K^−1^ (*V*_SE_/Δ*T* = 2.70 µV K^−1^). It should be noted that our zigzag structure consists of an odd number of wires. Thus, in the uniformly magnetized configuration, most of the ANE signals in the wires cancel each other out, and the small *V*_ANE_/Δ*T* value corresponds to the ANE thermopower originated from only one wire (recall the situation in Fig. 1b).

### Magneto-optical design of anomalous Nernst thermopile

Next, we utilized the AO-HDS to design the magnetization configuration locally, precisely, and reversibly and demonstrate the magneto-optical design of the anomalous Nernst thermopile. To obtain enhanced *V*_ANE_ signals with a reversible sign, two alternately reversed magnetization configurations with opposite **M** directions were established, as shown in Fig. 4e,f. The magnetization configuration was formed by alternately irradiating the wires uniformly magnetized along the − *z* (+ *z*) direction with right (**σ**^**+**^) (left (**σ**^**−**^)) circularly polarized laser light. Figure 4g shows the measured thermoelectric voltage signals in the two alternately reversed magnetization configurations. Compared with the thermoelectric voltage presented in Fig. 4c, there is a remarkable difference in the magnitude of the voltage and its sign is reversed by reversing the **M** direction. After removing the Seebeck offset estimated from Fig. 4d, we obtained the pure *V*_ANE_ contributions in the alternately reversed magnetization configurations. We found that the sign reversal of *V*_ANE_ depending on the **M** direction was symmetric and the magnitude of *V*_ANE_ obtained here was much larger than that in the uniformly magnetized film, consistent with our expectations (Fig. 4h). The ANE thermopower for the configuration in Fig. 4e (4f) was estimated to be 3.18 µV K^−1^ (− 3.31 µV K^−1^), which is approximately 10 times larger than that for the uniformly magnetized film (note again that all the thermoelectric voltage measurements were performed using the identical sample and that the signal for the uniformly magnetized film corresponds to the ANE thermopower originated from one wire). For the ideal case of the present zigzag structure with 13 wires, the enhancement ratio of *V*_ANE_ is expected to be 13 because *V*_ANE_ is proportional to the number of the wires. The smaller enhancement ratio obtained in our experiments is attributable to the non-100% AO-HDS. Nevertheless, the significant enhancement in *V*_ANE_ clearly demonstrates the validity of the magneto-optical design of the anomalous Nernst thermopile.

## Conclusion

We successfully demonstrated that, by combining the ANE with AO-HDS, the anomalous Nernst thermopile with a substantially enhanced thermoelectric output and a simple structure could be constructed. The AO-HDS enables local, precise, and reversible design of the magnetization configuration, potentially leading to orders of magnitude improvement of the ANE-induced thermoelectric voltage. As the anomalous Nernst thermopile designed using the AO-HDS consists of only a single material and does not require complicated microfabrication processes, it will pave the way toward developing thermoelectric devices with high flexibility, high mechanical endurance, low thermal resistivity, and low production costs. This concept is generalizable to all magnetic materials through exchange coupling with magneto-optical materials^28–34^, thus enabling the construction of anomalous Nernst thermopiles using materials having large *S*_ANE_.

## Methods

### Sample preparation

The sample used in this study was deposited on a single-crystalline Al_2_O_3_ (0001) substrate using the direct-current magnetron sputtering method at room temperature with a base pressure better than 5.0 × 10^−6^ Pa and a working pressure of 0.2 Pa. The optimized multilayer stack configuration was Al_2_O_3_ (0001) substrate/Ta (5)/Pt (5)/[Co (0.3) /Pt (0.7)]_4_/Pt (2.3) (unit in nm), where the Co/Pt multilayer film showed strong perpendicular magnetic anisotropy and high AO-HDS efficiency^35^. The bottom Ta/Pt layer improves the adhesion to the substrate and promotes the (111) texture of the Co/Pt multilayer, while the top Pt layer is necessary to avoid oxidation. The bottom Ta/Pt and top Pt layers should be as thin as possible to decrease shunting effects, which decrease the magnitude of the thermoelectric voltage induced by the ANE in the Co/Pt multilayer.

### Magneto-optical design of magnetization configuration

Before laser sweeping, the deposited [Co/Pt]_4_ film was microfabricated into a thermopile structure with 13 wires through photolithography using a lift-off process and subsequent Ar ion milling. The width and length of each wire are 0.2 mm and 5.0 mm, respectively. The patterned [Co/Pt]_4_ film was uniformly magnetized by applying an external magnetic field along the + *z* or − *z* direction. The magnetization configurations shown in Fig. 4e,f were then realized at room temperature by illuminating the wires with circularly polarized light via the AO-HDS. The laser pulses utilized here had a center wavelength of 514.0 nm, pulse duration of ~ 200.0 fs, and a repetition rate of 30.0 kHz. Excitation laser pulses with a diameter of 15.0 μm were focused on the sample surface and swept at a constant velocity of ~ 3.0 µm s^−1^. The typical laser power used was ~ 2.5 mW, and the corresponding laser fluence applied was 0.6 mJ cm^−2^ for the [Co/Pt]_4_ film. The magnetic domain patterns were subsequently imaged with a MOKE microscope (Fig. 3b–e).

### Thermoelectric voltage measurements

The thermoelectric voltage in the zigzag-shaped film was measured while applying a uniform temperature gradient perpendicular to the patterned wires (in the *x* direction) with a system similar to an in-plane temperature gradient generator used in ref. ^36^. Here, the sample was bridged between two Cu blocks separated by 8 mm. One of the Cu block can be heated by applying a charge current to a chip heater attached to the block, while the other block works as a heat bath. The voltage between the terminals connected to the end of the zigzag structure was measured using a microprobing system and a nanovoltmeter (2182A, Keithley) at room temperature and atmospheric pressure. The temperature difference, Δ*T*, between the terminals and the uniformity of the temperature gradient were confirmed by measuring the temperature distribution of the sample surface with an infrared camera after coating the surface with a black ink having high infrared emissivity (> 0.95). The magnitude of Δ*T* can be tuned by changing the charge current applied to the chip heater of the temperature gradient generator. With the out-of-plane magnetization easy axis of the [Co/Pt]_4_ film and the temperature gradient being along the *x* direction, the ANE-induced electric field was generated along the wires (*y* direction) according to the orthogonal relationship defined in Eq. (1). We confirmed that the magnitude of the background due to the SE (*V*_SE_/Δ*T* = 2.70 µV K^−1^) estimated by this procedure agreed well with the Seebeck coefficient of the [Co/Pt]_4_ film (2.72 µV K^−1^) measured with the Seebeck Coefficient/Electric Measurement System (ZEM-3, ADVANCE RIKO, Inc.). At each Δ*T* value, the thermoelectric voltage was measured five times and calculated the average value. In Fig. 4, the error bars of the thermoelectric voltages are smaller than the size of the data points, suggesting that the Δ*T* values and voltage measurements are stable.

### Hall measurements

To quantify the AO-HDS efficiency, we measured the out-of-plane magnetic field *H* dependence of the Hall resistance *R*_H_ in the [Co/Pt]_4_ film at room temperature (Fig. 2d). The films were patterned into a Hall bar structure with a cross area of 40 × 20 µm^2^ through photolithography and Ar ion milling. As shown in Fig. 2d, the *H*–*R*_H_ curve shows a rectangular hysteresis loop and the *R*_H_ values remain constant when *H* is greater than the coercive force, indicating that *R*_H_ is dominated by the anomalous Hall effect reflecting the **M** direction^37,38^. The AO-HDS efficiency was obtained by comparing the *R*_H_ values before and after illuminating the entire Hall bar with **σ**^−^ light at zero field, where the Hall bar was uniformly magnetized by applying an external field of *H* =  + 20 kOe before light illumination. The magnitude of the light-induced *R*_H_ change was approximately 91% of that of the *H*-induced *R*_H_ change due to the anomalous Hall effect.

## Data Availability

The data that support the findings of this study are available from the corresponding authors upon reasonable request.
